# MicroRNA Expression Profile Distinguishes Glioblastoma Stem Cells from Differentiated Tumor Cells

**DOI:** 10.3390/jpm11040264

**Published:** 2021-04-01

**Authors:** Sara Tomei, Andrea Volontè, Shilpa Ravindran, Stefania Mazzoleni, Ena Wang, Rossella Galli, Cristina Maccalli

**Affiliations:** 1 Research Department, Sidra Medicine, Doha PO26999, Qatar; ravindranshilpa@gmail.com (S.R.); cmaccalli@sidra.org (C.M.); 2 Unit of Immuno-Biotherapy of Melanoma and Solid Tumors, Division of Molecular Oncology, San Raffaele Foundation Scientific Institute, 20132 Milan, Italy; andrea.volo87@gmail.com; 3 Neural Stem Cell Biology Unit, Division of Neuroscience, San Raffaele Scientific Institute, 20132 Milan, Italy; stefania.mazzoleni@genenta.com (S.M.); galli.rossella@hsr.it (R.G.); 4 Infectious Disease and Immunogenetics Section (IDIS), Department of Transfusion Medicine, Clinical Center, and Center for Human Immunology (CHI) National Institutes of Health, Bethesda, MD 20892, USA; Ewang911@gmail.com

**Keywords:** glioblastoma, microRNAs, cancer, qPCR, cancer stem cells

## Abstract

Glioblastoma (GBM) represents the most common and aggressive tumor of the brain. Despite the fact that several studies have recently addressed the molecular mechanisms underlying the disease, its etiology and pathogenesis are still poorly understood. GBM displays poor prognosis and its resistance to common therapeutic approaches makes it a highly recurrent tumor. Several studies have identified a subpopulation of tumor cells, known as GBM cancer stem cells (CSCs) characterized by the ability of self-renewal, tumor initiation and propagation. GBM CSCs have been shown to survive GBM chemotherapy and radiotherapy. Thus, targeting CSCs represents a promising approach to treat GBM. Recent evidence has shown that GBM is characterized by a dysregulated expression of microRNA (miRNAs). In this study we have investigated the difference between human GBM CSCs and their paired autologous differentiated tumor cells. Array-based profiling and quantitative Real-Time PCR (qRT-PCR) were performed to identify miRNAs differentially expressed in CSCs. The Cancer Genome Atlas (TCGA) data were also interrogated, and functional interpretation analysis was performed. We have identified 14 miRNAs significantly differentially expressed in GBM CSCs (*p* < 0.005). MiR-21 and miR-95 were among the most significantly deregulated miRNAs, and their expression was also associated to patient survival. We believe that the data provided here carry important implications for future studies aiming at elucidating the molecular mechanisms underlying GBM.

## 1. Introduction

Glioblastoma is the deadliest malignant intracranial tumor in adults. In the United States its annual incidence is 3.2 cases per 100,000 people [1,2], while in Europe the incidence is 3–5 cases per 100,000 people [3]. Its progression is accompanied by a rapid spread, an infiltrative growth and high cellular heterogeneity [4,5]. The current management of Glioblastoma (GBM) patients includes surgical resection, radiotherapy, chemotherapy and tumor treating fields (TTFields) [4,6]. Among the chemotherapeutic agents, temozolomide (TMZ) is the most common alkylating agent employed in the clinical management of GBM patients. However, GBM has been shown to acquire resistance to TMZ, thus explaining GBM recurrence [4,6]. GBM prognosis is generally poor and the median survival is only 14 months, while the 5-year survival rate is unfortunately between 5%–10% [7,8,9,10]. Overall, 90% of GBM originate de novo while the remaining 10% arise from lower-grade glioma [11,12]. Although the exact etiology of GBM is still being explored, it has been reported that ionizing radiation at high-dose and rare genetic disorders could facilitate the onset of GBM [13]. Advancements on the molecular understanding of GBM onset and progression are warranted to help the clinical management of GBM.

The most important molecular biomarkers for GBM include the methylation status of O-6-methylguanine-FNA methyltransferase (MGMT) promoter, and the mutational status of the isocitrate dehydrogenase 1 and 2 (IDH1, IDH2). When the MGMT promoter is found methylated, GBM has been reported to have a better outcome [13]. Furthermore, IDH1 and IDH2 mutations have been found correlating with a less aggressive GBM phenotype [1]. Nevertheless, the shortage in molecular biomarkers for GBM underscores the importance to implement new studies for the identification of novel biomarkers, such as microRNAs (miRNAs). MiRNAs are small non-coding RNAs 18–25 nucleotides long. They are transcribed by the RNA polymerase II and processed into their mature form through a series of steps involving Drosha/DGCR8 complex, for the generation of pre-miRNAs, and Dicer, which generates the mature form of miRNAs. Several technologies are now being applied for the study of miRNAs [14]. MiRNAs recognize the complementary sequences in the 3′ untranslated regions (3′UTR) of given transcripts to cause their degradation or translational repression [15,16]. Different types of cancers display miRNA expression dysregulation through different mechanisms, encompassing genomic variations in miRNA encoding genes, deregulation of miRNA transcription, epigenetic mechanisms and disruption of the miRNA synthesis machinery [17,18]. Several miRNAs have been shown to be dysregulated in GBM as compared to normal brain samples [19].

Increasing scientific evidence has pointed to the existence within tumor lesions of a cell subpopulation with stem-like properties, defined “cancer stem cells” (CSCs). GBM CSCs have the ability to self-renew, differentiate multi-potentially in the three lineages of the central nervous system (CNS) and to give rise to tumor when transplanted experimentally [20,21,22,23]. Additionally, CSCs have been shown to resist radiotherapy and chemotherapy, thus inducing continued proliferation and possibly mediating tumor recurrence [4,24,25,26,27]. Moreover, it has been shown that CSCs display intrinsic ability to protect themselves from both natural and adaptive immune responses [22,28,29,30,31]. Targeting GBM CSCs represents an ideal approach to treat GBM and overcome tumor recurrence. Despite the fact that there exists good epidemiological evidence of GBM, the molecular mechanisms underlying its onset and progression are only recently starting to be elucidated.

Currently, studies aimed at evaluating miRNA expression in GBM CSCs are scarce. This study aims at identifying miRNAs potentially explaining GBM CSC properties and their potential immunological resistance. We have employed an array-based quantitative real-time PCR approach and evaluated the differences in the expression of miRNAs in pairs of autologous CSCs and differentiated tumor cells obtained from the same GBM patients. Finally, we have also performed functional pathway analysis and interrogated The Cancer Genome Atlas (TCGA) for validation purposes.

## 2. Results

### 2.1. MiRNAs Differentially Expressed between CSCs and Autologous Differentiated Cells

The characterization of the profile of miRNAs in different CSC lines (*n* = 11) vs. differentiated bulk tumor cells (*n* = 4) in GBM was initially performed utilizing custom-made arrays for the detection of 713 human, mammary and viral miRNAs. Class comparison between the two groups identified 20 miRNAs differentially expressed at a significance level of *p* < 0.01 (Supplementary Figure S1A). This analysis was refined by utilizing only the 3 CSCs and their 3 corresponding autologous differentiated cells (from here on denominated “FBS” as they were grown in fetal bovine serum (FBS); Supplementary Figure S1B).

**Figure 1:**
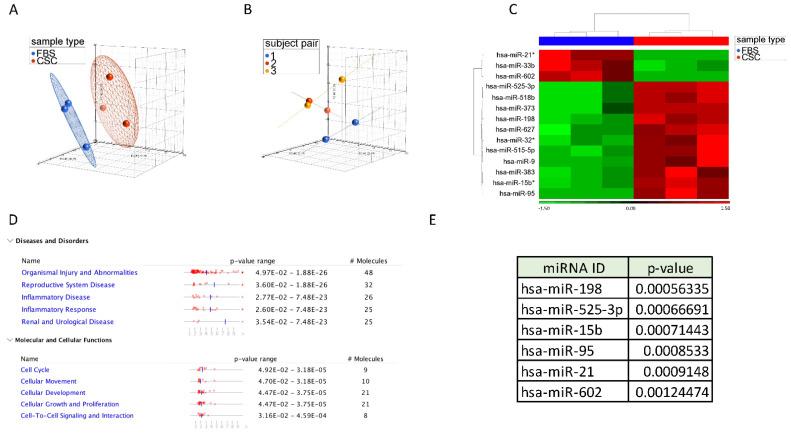
Principal Component Analysis (PCA) of CSC (in red) and FBS (in blue) samples based on the complete miRNA expression data set (**A**). Principal Component Analysis (PCA) of the samples according to their individual pair (**B**). Hierarchical clustering of the 14 significantly differentially expressed miRNAs (*p* < 0.005); miRNAs marked with the asterisk (*) correspond to a less abundant form (**C**). Functional Interpretation analysis of 67 differentially expressed miRNAs at a statistical level of *p* < 0.05 (**D**). List of the 6 most significant differentially expressed miRNAs between CSC and FBS samples (**E**).

We then validated the differential profile of CSC lines vs. FBS cell lines through quantitative Real-Time PCR (qRT-PCR) of 704 mature miRNAs, annotated by the Sanger miRBase Release 14. To test whether the assignment of the samples to the CSC and FBS groups would have translated in a different miRNA expression, we have applied principal component analysis (PCA) to the complete miRNA data set. The assignment of the individual samples to the CSC and FBS groups predicted their distribution in a three-dimensional space suggesting that CSCs and FBS samples displayed a differential expression of the miRNAs included in the complete dataset (Figure 1A). Interestingly, samples belonging to the same individual pairs clustered closely to each other compared to the other samples (Figure 1B), suggesting that the intra-individual miRNA expression variability was lower than the inter-individual miRNA expression variability.

The miRNA expression data were next used to identify miRNA discriminating the CSC and FBS samples. At a significance level of *p* < 0.05, 67 miRNAs resulted differentially expressed between CSC and FBS samples. When using a more stringent *p*-value (*p* < 0.005), 14 miRNAs were differentially expressed, with miR-21, miR-33b and miR-602 being up-regulated in the FBS cells compared to CSCs, while the remaining miRNAs (miR-525, miR-518, miR-373, miR-198, miR-627, miR-32, miR-515, miR-9, miR-383, miR-15 and miR-95) were up-regulated in CSCs compared to their paired FBS cells (Figure 1C).

The functional interpretation by Ingenuity Pathway Analysis (IPA) of the 67 miRNAs differentially expressed at *p* < 0.05 revealed that they were associated with inflammatory disorders as well as functions relating to the cell cycle, cell movement, cell development and cell proliferation (Figure 1D).

### 2.2. Functional Interpretation Analysis and Interrogation of miRDB and TargetScan

**Figure 2:**
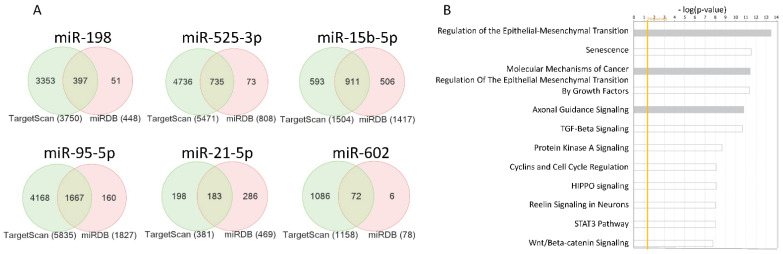
Venn diagrams of the target genes identified by TargetScan and miRDB for the six most significant miRNAs (**A**). Top canonical pathway identified on IPA from the list of the target genes in the intersections of the Venn diagram of the six most significant miRNAs (**B**). The significance values (*p*-value of overlap) for the canonical pathways are calculated by the right-tailed Fisher’s Exact Test. The x-axis displays the -log of the *p*-value. The orange line indicates the significance threshold. Gray bars indicate pathways for which no prediction of activation or inhibition can be made due to insufficient evidence in the Knowledge Base for confident activity predictions across datasets. White bars indicate pathways with z-scores at or very close to 0.

Out of the 14 miRNAs significantly differentially expressed between GBM CSCs and their autologous differentiated cells, we have selected the top ranking 6 miRNAs and queried them for further analyses (Figure 1E). We have used two available online miRNA resources to interrogate miRNA target genes, namely TargetScan and miRDB [32,33,34]. With the assumption that target genes identified by both platforms for the same miRNA would be more indicative of a real miRNA-target gene interaction, we have generated Venn diagrams for the six most significant miRNAs (Figure 2A). The target genes in the intersection of the six Venn diagrams were combined in a unique list and submitted for functional interpretation analysis to Ingenuity Pathway Analysis (IPA). The analysis revealed an enrichment of genes related to neuronal signaling, cell differentiation and cell cycle regulation as well as pathways associated with the molecular mechanisms of cancer, the regulation of epithelial-mesenchymal transition (EMT) and the Hippo pathway. Several of these pathways were relevant for “stemness” properties. These findings support the role of the 6 top-ranking miRNAs and their target genes in regulating GBM CSC biology (Figure 2B). Nevertheless, the list of the top-ranking canonical pathways included also immune-related signaling pathways, such as the Transforming Growth Factor (TGF)-β signaling and STAT3 signaling pathways, suggesting a potential role of these miRNAs and their target genes in regulating immune-related functions. The functional interpretation analysis was also carried at the individual miRNA level (Supplementary Figure S2). Such analysis highlighted that additional immune-related pathways (e.g., Interleukin (IL)-1, Interferon (IFN), NF-kB, Toll-like receptor and T cell proliferation or exhaustion) could be regulated by the miRNAs differentially expressed in CSCs vs. FBC cell lines.

### 2.3. Validation on the TCGA Dataset

We interrogated the role of the six most significant differentially expressed miRNAs using the Glioblastoma Bio Discovery Portal (GBM-BioDP) [35]. GBM-BioDP is an online visualization platform that allows the access of miRNAs differentially expressed across GBM samples from the TCGA database [36]. The genomic profiling of GBM from TCGA has led to the definition of four GBM subtypes, namely the classical, the mesenchymal, the neural and the proneural subtypes. Such subtypes might develop through different and independent molecular mechanisms [36]. Among the four subtypes, the mesenchymal one displays a higher necrosis percentage and inflammation features, potentially due to the activation of the NF-kB signaling pathway. The mesenchymal subtype also displays poor prognosis as compared to the other subtypes [37].

**Figure 3:**
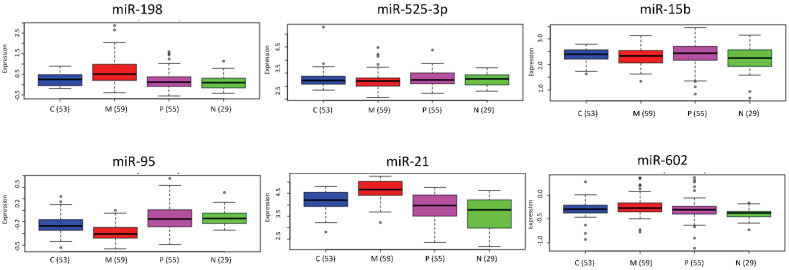
Boxplots of the six most significant miRNAs expression distribution in four subtypes of Glioblastoma (GBM) of The Cancer Genome Atlas (TCGA) patients.

To further explore whether the significantly differentially expressed miRNAs between CSCs and differentiated cells found in this study were related to GBM transcriptional subgroups, we have interrogated the six most significantly deregulated miRNAs in GBM against GBM-BioDP. Among them, we found that miR-95 and miR-21 expression was significantly different in the mesenchymal group as compared to the other groups (Figure 3). Specifically, miR-95, which we found upregulated in CSCs, was significantly downregulated in the mesenchymal group compared to the proneural and neural subtypes (*p* = 0.017 and *p* = 0.008, respectively), while miR-21, which was downregulated in CSCs, resulted upregulated in the mesenchymal group as compared to the proneural and neural subtypes (*p* = 0.013 and *p* = 0.001, respectively).

**Figure 4:**
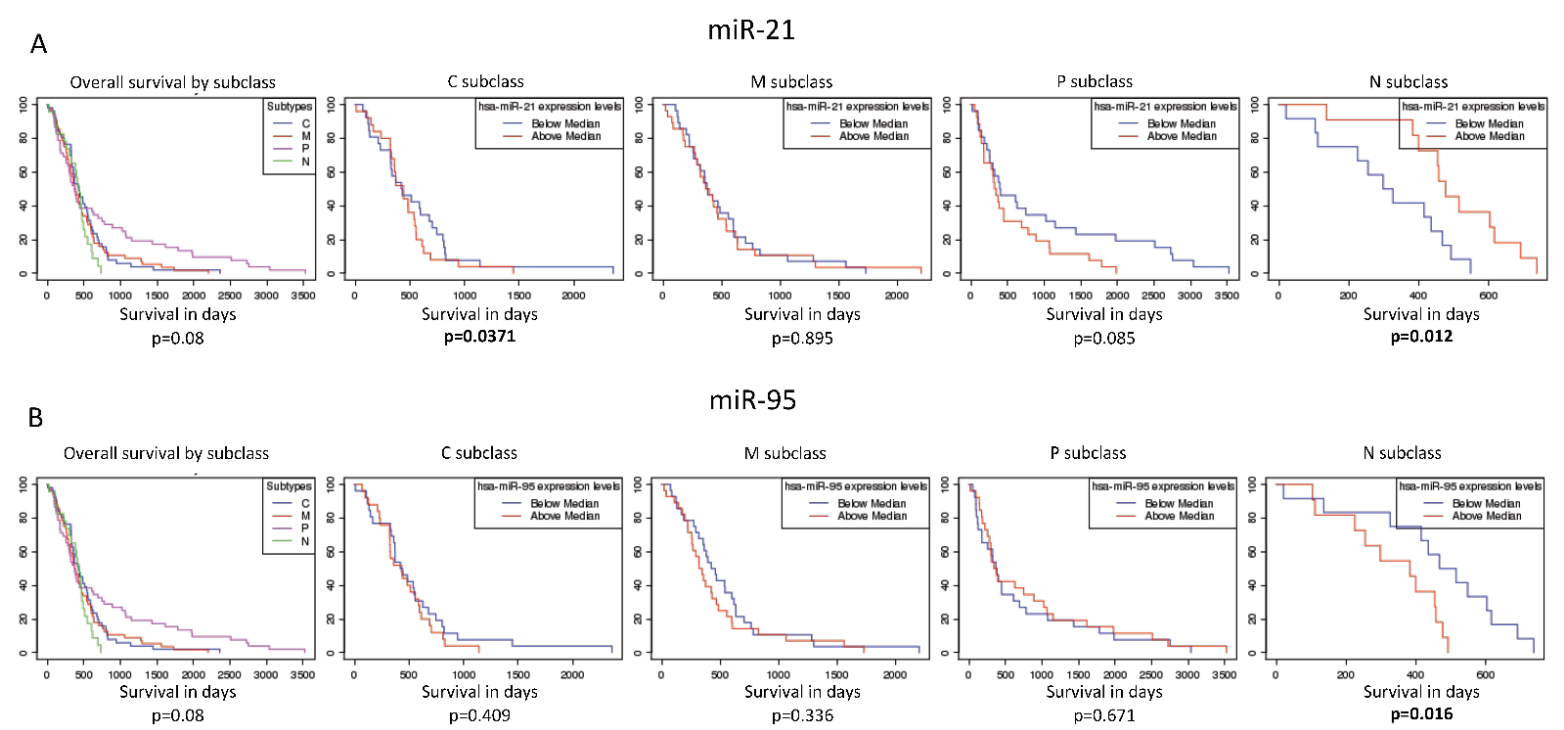
Kaplan–Meier analysis of miR-21 (**A**) and miR-95 (**B**) in the four subtypes of GBM of the TCGA patients.

We further questioned whether the expression of miR-95 and miR-21 was associated to survival outcome. The Kaplan–Meier analysis obtained from GBM-BioDP showed an association of a higher expression of miR-21 and a lower expression of miR-95 to a better survival for the neural subtype (*p* = 0.012 and *p* = 0.016, respectively), although no significant association was found for the other subtypes (Figure 4). Genes known to be the targets of miR-95 and miR-21 have also been interrogated against GBM-BioDP. When their expression across the TCGA GBM subtypes was inversed to the one reported for their corresponding miRNA, we assumed this observation to be more indicative of a real miRNA-target gene interaction. The analysis of miR-95 targets revealed an inversed expression of several genes involved in EMT (HGF and MAP2K3), STAT3 signaling (IL10RA) and in the role of macrophages, fibroblast, endothelial cells (TGF-β1 and IL-15) (Supplementary Figure S3). The analysis of miR-21 targets revealed an inversed expression of several genes involved in EMT (PIK3R1 and GAB1), TGF-β signaling (BMPR2) and in IL-1 signaling (GNAZ and MAP2K4) (Supplementary Figure S4).

## 3. Discussion

GBM remains one of the most lethal solid tumors [38]. Despite the scientific efforts to understand GBM pathogenesis over the last decade, GBM prognosis remains poor, highlighting the challenges in the clinical management of this cancer. GBM displays high level of intratumoral heterogeneity as well as cellular differentiation hierarchy. In fact, increasing scientific evidence supports the existence of a subpopulation of CSCs in GBM with self-renewal capabilities.

MiRNAs are emerging as critical regulators of proliferation and differentiation, and some of them have been shown to carry an important role in CSCs [38]. In this study we questioned the role of miRNA differential expression in CSCs and their corresponding autologous differentiated cells. The PCA analysis on the complete dataset gave a clear separation of the CSCs and the differentiated cells, suggesting that the complete miRNA data set was able to discriminate the two groups, although sample pairs belonging to the same individual clustered more closely compared to the others (Figure 1A,B). The class comparison between CSCs and their autologous differentiated cells identified 67 differentially expressed miRNAs at a significance level of *p* < 0.05. Functional interpretation analysis revealed several cellular functions related to cell growth and proliferation, as expected. When we refined the list to only 14 most significant miRNAs, we found that the majority of miRNAs (*n* = 11) was upregulated in the CSCs as compared to their differentiated counterparts. Among the miRNAs up-regulated in CSCs vs. FBS tumor cells, miR-515, miR-15b and miR-198 resulted as regulators of glioma associated signaling. MiR-95, miR-21 and miR-627 represent key regulators of the EMT signaling that has been shown to be one of the initial mechanisms of CSCs formation [39,40,41]. Additionally, miR-9, miR-32, miR-383, miR-518, miR-627 and miR-602 are involved in mechanisms regulating the expression/activation of PTEN, p53, Hippo, stem cell pluripotency and Notch signaling that are among the principal pathways involved in stemness-associated features [42,43,44,45].

GBM CSCs are endowed with superior self-renewal and tumorigenic ability [20] as compared to the differentiated bulk tumors that might be regulated by the differential miRNA profile. In particular, the aforementioned miRNAs could represent relevant modulators of the expression of target genes involved in signaling pathways associated with stemness properties (e.g., EMT, Notch, Hippo, p53, PTEN etc.).

CSCs also represent the component of tumors responsible of resistance to chemotherapy and radiotherapy [26,27,46]. MiR-383 and miR-525 were also found up-regulated in CSCs vs. FBS cells, and they were reported to modulate the signaling associated with cell survival and DNA damage that can occur following chemotherapy or radiotherapy [47,48].

To gain further insights on the differential miRNA expression between CSCs and their differentiated paired cells, we looked into the target genes of the six most significant miRNAs. Target genes identified by TargetScan and miRDB of the six most significant miRNAs (Figure 2A) were combined in a unique list and queried for functional interpretation analysis, which identified several GBM-related pathways among the top-ranking canonical pathways (Figure 2B). Additionally, immune-related pathways were also identified, including TGF-β and STAT3 signaling, supporting a potential immune-related role of the six most significant miRNAs and their target genes.

Recurrent genomic alterations in GBM have been largely catalogued by TCGA Network. Based on these alterations, GBM samples have been grouped into four main subtypes, namely the classical, the neural, the proneural and the mesenchymal subtypes, the latter being the subgroup with worst prognosis [36,37]. The mesenchymal subgroup has also been characterized by a higher percentage of necrosis and inflammation features, potentially due to the activation of the NF-kB signaling pathway [37]. When we looked into the expression of the six most significant miRNAs in the TCGA dataset, we found that miR-21 was significantly upregulated, and miR-95 significantly downregulated in the mesenchymal group. Survival analysis also showed that miR-21 upregulation and miR-95 down-regulation were associated with better survival in the neural subtype only. MiR-21 is a well-studied miRNA, which acts as an oncomir and associates with a malignant phenotype [4,49]. It has been very recently reported being significantly downregulated in GBM CSCs compared to astrocytes [11] and has already been shown to associate with a more differentiated phenotype [50,51]. The primary CSCs utilized in the present manuscript have been assessed molecularly and resulted to belong mostly to the proneural transcriptional subgroup [52,53]. However, the mechanisms behind the association of the aforementioned miRNAs need to be further elucidated. MiR-95 has been reported to promote growth in colorectal, pancreas, prostate and breast cancer, but also to have anticancer activity in hepatic, brain and neck cancer [54,55,56]. In glioma, it was reported that the downregulation of miR-95 decreases the proliferation and invasion while promoting apoptosis of glioma cells [57]. Nevertheless, evidence on the role of miR-95 in GBM is scarce. We believe that our study adds important evidence to the role of miR-95 in GBM.

We acknowledge that to further elucidate the role of miR-95 and miR-21 in particular pathways, it is recommended to establish a knock-down of miRNA and explore the expression of the related downstream targets.

The characterization of the immunological profile of CSCs highlighted a general suboptimal immunogenic potency and susceptibility to cell-mediated immune responses [22,28,29,30,31]. Our group has previously reported that GBM-CSCs display a differential immune profile, including HLA molecules, soluble cytokines and growth factors compared to FBS cells, rendering these cells resistant to T cell-mediated responses [22]. Gene and protein expression determination showed a differential profile in the detection of TGF-β1, TGF-β2, IL-6 and IL-8 in GBM CSCs vs FBS cell lines [22]. Of note miR-95 expression inversely correlates with the expression of the target gene TGF-β1, as shown in Supplementary Figure S3. Therefore, the overexpression detected in GBM-CSCs of this miRNA could be responsible of the down-modulation of TGF-β1 observed in CSCs vs FBS cell lines [22]. Moreover, genes associated with IFN and Tumor Necrosis Factor (TNF) signaling were down-modulated in CSCs vs. FBS cells, including targets of miR-383, miR-627 or miR-525, respectively, that are overexpressed in CSCs (Figure 1) [22]. Conversely, the JAK-STAT signaling pathway was up-regulated in cells with stemness properties compared to differentiated cells [22]. Moreover, JAK-STAT signaling can be modulated by miR-9, miR-15, miR-32, miR-95, miR-373 and miR-515 that were differentially expressed in CSCs as compared to their differentiated cells. The molecular make-up of CSCs could be orchestrated by the pattern of miRNAs detected in these cells. STAT3, TGF-β and IFN signaling can be regulated by multiple miRNAs, such as miR-21, miR-32, miR-95, miR-585, miR-373, miR-383 and miR-627. CSCs could efficiently modulate both natural and adaptive immune responses in their relationship with the crosstalk with tumor microenvironment [29,30,58].

Interestingly, the levels of the immune checkpoint Ligand of Programmed Death Ligand 1 (PD-L1) has been reported to be modulated either directly by miR-33 [59] or indirectly through the regulation of the levels of PTEN by miR-21 [60]. PD-L1 has been previously reported to be expressed at high level in GBM-CSCs and, possibly being one of the molecules responsible for the impairment of T-cell mediated immune responses against these cells [22]. Our observations in this study suggest the importance of miR-33 and miR-21 as key regulators of the immunoregulatory properties of GBM-CSCs.

MiR-32 and miR-95, through the modulation of RAC or macrophage-, endothelial- and fibroblast-associated signaling pathways, respectively, could be among the key regulators of this phenomenon. Taken together, the analyses of the miRNA profile in pairs of CSCs vs. the differentiated tumor cells led to the identification of key regulators of signaling involved in stemness and immunological properties of GBM tumor initiating cells. The obtained results corroborate the hypothesis that the profile of miRNAs in CSCs may mediate the resistance of CSCs to immune responses.

We believe that our study provides additional information on the role of GBM CSCs. Previous studies have investigated the role of miRNAs in GBM CSC, nevertheless they employed CSC samples rather than autologous pairs [61,62,63].

The role of specific miRNAs in GBM CSCs has started being explored [64]. However, further functional investigations are warranted to demonstrate the link between the miRNAs identified in our study and the functions of CSCs. Moreover, additional techniques, such us in situ hybridization and other staining analyses, might help in corroborating our findings. We believe that this evidence paves the way toward the identification of tools that can modulate miRNA expression to optimize the efficacy of targeting GBM CSCs with immunotherapy.

In conclusion, we show here that several miRNAs are associated with GBM CSCs. MiR-21 and miR-95 are among the most significant differentially expressed miRNAs that carry implications on GBM molecular profiling and patient survival. Other miRNAs have been identified as potential regulators of the immunogenicity of CSCs. Additional analyses on larger cohorts are necessary to validate our findings and elucidate the molecular mechanisms behind GBM CSCs in further details.

## 4. Materials and Methods

### 4.1. Cell Culture and RNA Isolation

Cancer cells were of human origin. Tumor specimens were collected from *n* = 11 GBM patients admitted at the San Raffaele Hospital Scientific Institute, Milan, Italy. The study was approved by the IRB and patients were enrolled in the study upon signature of the informed consent. GBM cell lines, both CSCs and bulk tumor cells (denominated FBS) were established in vitro from fresh GBM lesions as previously described [22]. Briefly, GBM CSCs were cultured in vitro in the form of neurospheres in the presence of DMEM/F12 medium containing 20 ng/mL of epidermal growth factor (EGF) and fibroblast growth factor (FGF2) (Peprotech, Rocky Hill, NY, USA) plus additives as described in Galli et al. [20]. Primary cells were plated in 25 cm^2^ tissue culture flasks at a clonal density of 2500–5000 cells/cm^2^. When enough tumor tissue was available, a portion of the cells obtained from the enzymatic digestion was plated in the presence of RPMI 1640 supplemented with 10% FBS (Biowittaker, Lonza, Treviglio, Italy) to generate the aforementioned differentiated tumor cells. Early-in vitro passage (*n* = 10–15) cultures were used for all the experiments.

Total RNA was isolated from 3 × 10^6^ cells using miRNeasy minikit (QIAGEN, Hilden, Germany), according to the manufacturer’s protocol. RNA quantity and quality were assessed using Nanodrop Spectrophotometer (Thermo Fisher Scientific, Waltham, MA, USA) and Bioanalyzer (Agilent Technologies, Carlsbad, CA, USA). Samples were evaluated according to their RIN (RNA Integrity Number).

### 4.2. Array Screening of miRNAs

A miRNA probe set was designed using mature antisense miRNA sequences (Sanger data base, version 9.1) consisting of 827 unique miRNAs from human, mouse, rat and virus plus two control probes. The probes were 5′ amine modified and printed in duplicate on CodeLink activated slides (General Electric, GE Health, Midland Park, NJ, USA) via covalent bonding at the Infectious Disease and Immunogenetics Section of the Department of Transfusion Medicine (DTM) (Clinical Center, NIH, Bethesda, MD, USA). Four μg of total RNA isolated by using Trizol reagent (Invitrogen, Gaithersburg, MD, USA) were directly labelled with miRCURY™ LNA Array Power Labelling Kit (Exiqon, Woburn, MA, USA) according to manufacturer’s procedure. The total RNA from the Epstein–Barr virus (EBV)-transformed lymphoblastoid cell line was used as the reference for the miRNA expression array assay. The test sample was labelled with Hy5 and the reference with Hy3. After labelling, the sample and the reference were co-hybridized to the miRNA array at room temperature overnight in the present of blocking reagents as previously described and the slides were washed and scanned by GenePix scanner Pro 4.0 (Axon, Sunnyvale, CA, USA). Resulting data files were analyzed using Partek Genomics Suite. Hierarchical cluster analysis and TreeView [65] software were used for visualization [66].

### 4.3. Quantitative Real-Time PCR (qRT-PCR) of miRNAs

The quantitative determination of the profile of miRNAs was performed with the RT2 miRNA PCR Array System (QIAGEN). MiRNA Sequence Specific Assays include one universal primer and one gene-specific primer for each miRNA sequence. This kit includes PCR Arrays to determine through a SYBR^®^ Green real-time PCR detection system, the expression of 704 mature miRNAs annotated by the Sanger miRBase Release 14. The panel included SNORD 48, 47, and 44 and U6 as housekeeping assays that were used to normalize the qRT-PCR array data. The kit also included two RNA and PCR quality controls to test the efficiency of the RT2 miRNA first strand kit and efficiency of the polymerase chain reaction, respectively.

Two-hundred ng of small RNA were used for reverse transcription and the first-strand cDNA was synthesized with the RT2 miRNA First Strand Kit according to the manufacturer’s instructions (cat. no. 331401). First strand cDNAs were mixed with RT2 SYBR Green qPCR Mastermix and the experimental cocktail was added to each corresponding well of the PCR arrays. PCR conditions were: 10 min at 95 °C and 40 cycles of: 15 s at 95 °C, 30 s at 60 °C and 30 s at 72 °C. The reaction was run on the ABI 7900 HT thermal cycler (Applied Biosystems). A melting curve analysis was performed to check assays’ specificity. Samples were run in duplicates. The geometric mean of the housekeeping genes was subtracted from the Ct (Cycle threshold) values of each sample to give a delta Ct value that corrects for different sample amounts. Delta Ct values were then transformed into the negative delta Ct values and used to calculate the 2^-delta Ct.

### 4.4. Data Analysis

Principal component analysis (PCA) was applied for visualization when relevant. All the graphical analyses were performed using Partek Genomic Suite tool (Partek, St. Louis, MO, USA). MiRNA expression class comparison between the CSC and FBS cell lines was based on the analysis of variance (ANOVA). All statistical tests were two-sided. *p*-values lower than 0.05 were considered statistically significant. Functional interpretation analysis was performed using Ingenuity Pathway Analysis (IPA) tools 3.0 (QIAGEN), which transforms large data sets into group of relevant networks including direct and indirect relations among genes based on known interactions established from the literature. Heat-maps are presented based on Partek visualization program.

The TCGA dataset was interrogated for validation purposes. The TCGA data was accessed by using the GBM-BioDP [35]. MiRNA and target genes expression profiles were queried and visualized based on known molecular subtypes [36].

Two-sided *t*-tests comparing the miRNA expression levels between subtypes were performed and their significance values were reported. Kaplan–Meier survival rate analysis was also performed. The dataset included 196 TCGA patients of the following subtypes according to Verhaak et al. [36]: classical (*n* = 53), mesenchymal (*n* = 59), proneural (*n* = 55), and neural (*n* = 29). The mRNA expression of specific miRNA target genes was evaluated using the experiments in the 3-Platform aggregates.

## 5. Conclusions

Despite the recent scientific efforts to understand GBM etiology and pathogenesis, GBM remains a complex disease and one of the most lethal tumors. Given the low survival rate, it is of primary importance finding biomarkers that could improve the clinical management of GBM patients. This article provides evidence that a group of specific miRNAs can explain the stemness properties and the immunological profile of CSCs in GBM. A further validation of our findings is warranted through studies employing larger cohorts.

## Data Availability

The data presented in this study are available on request from the corresponding author.

## Supplementary Materials

The following are available online at https://www.mdpi.com/article/10.3390/jpm11040264/s1, Figure S1: Hierarchical clustering of the 20 significantly differentially expressed miRNAs in *n* = 11 CSCs vs. *n* = 4 FBS cell lines identified through in house made arrays (*p* < 0.01) (A). Hierarchical clustering of the significantly differentially expressed miRNAs in 3 pairs of autologous CSCs and FBS cell lines identified through in house made arrays (*p* < 0.01) (B). Figure S2: Canonical pathways by individual miRNAs. These pathways were identified by querying the target genes (at the intersection of miRDB and TargetScan Venn diagrams) for each individual miRNA of the 14 significant miRNAs (*p* < 0.005). Both the 5p and 3p strands were included. The color legend indicates pathways associated with: 1. Cancer development and stemness features (green); 2. Immune-related functions (orange); 3. Pathways with pro-tumoral and immunological functions (yellow); 4. Cell regulation function that can be aberrantly modulated in cancer (violet); 5. Others (white). Figure S3: Boxplots of the distribution of the expression of miR-95 targets in the four subtypes of GBM of the TCGA patients. We have reported in brackets the molecular pathways associated to the individual target genes. Figure S4: Boxplots of the distribution of the expression of miR-21 targets in the four subtypes of GBM of the TCGA patients. We have reported in brackets the molecular pathways associated to the individual target genes.

## Abbreviations

ANOVA	Analysis of Variance
CNS	Central Nervous System
CSC	Cancer Stem Cells
Ct	Cycle threshold
EMT	Epithelial Mesenchymal Transition
EGF	Epidermal Growth Factor
FBS	Fetal Bovine Serum
FGF2	Fibroblast Growth Factor
GBM	Glioblastoma Multiforme
GBM-BioDP	Glioblastoma Bio Discovery Portal
HK	Housekeeping genes
IDH1	isocitrate dehydrogenase 1
IDH2	isocitrate dehydrogenase 2
IL	Interleukin
IFN	Interferon
IPA	Ingenuity Pathway Analysis
MGMT	O-6-methylguanine-FNA methyltransferase
miRNA	micro-RNA
NCI	National Cancer Institute
PCA	Principal Component Analysis
PD-L1	Ligand of Programmed Death Ligand 1
qRT-PCR	quantitative Real Time Polymerase Chain Reaction
RIN	RNA Integrity Number
TCGA	The Cancer Genome Atlas
TGF	Transforming Growth Factor
TNF	Tumor Necrosis Factor
TTFields:	Tumor Treating Fields
TMZ	Temozolomide
UTR	untranslated region
